# The Effects of Messaging on Long COVID Expectations: An Online Experiment

**DOI:** 10.1037/hea0001230

**Published:** 2022-09-15

**Authors:** Freya Mills, Jaskiran Kaur Bhogal, Amelia Dennis, Cristina Spoiala, Joanna Milward, Sidra Saeed, Leah Ffion Jones, Dale Weston, Holly Carter

**Affiliations:** 1 Behavioural Science and Insights Unit, UK Health Security Agency, Salisbury, United Kingdom; 2 Data, Analytics and Surveillance, UK Health Security Agency, London, United Kingdom

**Keywords:** long COVID, COVID-19, expectations, communication

## Abstract

***Objective:*** We examined whether varying information about long COVID would affect expectations about the illness. ***Method:*** In October 2021, we conducted a 2 (Illness Description: long COVID vs. ongoing COVID-19 recovery) × 2 (Symptom Uncertainty: uncertainty emphasized vs. not emphasized) × 2 (Efficacy of Support: enhanced vs. basic support) between-subjects randomized online experimental study. Participants (*N* = 1,110) were presented with a scenario describing a positive COVID-19 test result, followed by one of eight scenarios describing a long COVID diagnosis and then completed outcome measures of illness expectations including: symptom severity, symptom duration, quality of life, personal control, treatment control, and illness coherence. ***Results:*** We ran a series of 2 × 2 × 2 ANOVAs on the outcome variables. We found a main effect of illness description: individuals reported longer symptom duration and less illness coherence when the illness was described as long COVID (compared to ongoing COVID-19 recovery). There was a main effect of symptom uncertainty: when uncertainty was emphasized, participants reported longer expected symptom duration (*p* < .001), less treatment control (*p* = .031), and less illness coherence (*p* < .001) than when uncertainty was not emphasized. There was a main effect of efficacy of support: participants reported higher personal control (*p* = .004) and higher treatment control (*p* = .037) when support was enhanced (compared to basic support). ***Conclusions:*** Communications around long COVID should avoid emphasizing symptom uncertainty and aim to provide people with access to additional support and information on how they can facilitate their recovery.

Long COVID, also referred to as Post-COVID-19 syndrome, Postacute COVID-19, and ongoing symptomatic COVID-19, describes the symptoms of COVID-19 that develop during or after a COVID-19 infection, which continue for more than 4 weeks and cannot be explained by an alternative diagnosis (National Institute for Health and Care Excellence, 2020b). Common symptoms include fatigue, shortness of breath, muscle ache, and difficulty concentrating, with evidence suggesting that such symptoms may be experienced by up to 2.0% of the general population (Office for National Statistics, 2021), affecting day-to-day activities in 64% of those affected (Office for National Statistics, 2021). Similar postviral conditions have been observed following other coronavirus infections, such as SARS and MERS, as well as influenza and Ebola (Islam et al., 2020; Poenaru et al., 2021).

## Impact of Illness Expectations on Health Outcomes

While research is ongoing to understand the biological basis for long COVID, it is well-recognized that psychological factors can also affect physical outcomes associated with a variety of different illnesses (Pennebaker & Skelton, 1978; Pennebaker, 2012); it is therefore essential to consider psychological processes alongside biological factors. One such psychological process is one’s expectations of illness progression (Pagnini, 2019). Illness expectations can include expected symptom severity, symptom duration, illness coherence, quality of life, treatment control, and personal control. It is important to assess illness expectations as negative illness expectations have been shown to predict slower recovery rates, increased disability, worse physical symptoms, slower return to work, and reduced well-being (Broadbent et al., 2008; Karademas, 2006; McCarthy et al., 2003; Park et al., 2020, 2016; Petrie & Weinman, 2006). The Common-Sense model of Self-Regulation suggests that illness expectations can be formed by an individual’s representation of their symptoms and illness, which is determined by factors such as illness description and label, the actual and anticipated symptoms and consequences, and the extent to which symptoms can be managed (see Table 1 for definitions), all of which are unclear and continually changing for those with long COVID (Ladds et al., 2020).[Table tbl1]

## Factors Influencing Illness Expectations

### Illness Description

One factor associated with negative expectations is the way in which an illness is described (Petrie et al., 2018). It has been suggested that novel illness descriptions should be avoided until scientific evidence and medical consensus supports them (Clauw et al., 2003), and existing case descriptions should be used in the meantime as the label given to an illness can influence perceptions and behaviors in relation to the illness. Indeed, in research concerning hypothetical scenarios relating to polycystic ovary syndrome and gastroesophageal reflux disease, individuals were more likely to respond to their diagnosis (e.g., through medicine or further tests) when symptoms were given a diagnostic label than when they were not (Scherer et al., 2013; Copp et al., 2017). Also, the label given to symptoms can influence perceived symptom severity and participants’ expected well-being (Copp et al., 2017; Petrie et al., 2018). Furthermore, labeling a new syndrome (such as long COVID) could increase illness identity (the degree to which a chronic illness becomes part of one’s identity), which may in turn reduce quality of life (Moss-Morris et al., 1996).

### Symptom Uncertainty

Another key factor that has been shown to increase negative expectations is illness uncertainty, particularly in relation to uncertainty about likelihood and severity of symptoms. Uncertainty surrounding one’s illness has been linked to increased symptom severity, lack of personal control, decline in mental health, and diminished quality of life, among other outcomes (Wright et al, 2009). The Uncertainty in Illness Theory suggests that uncertainty is related to four factors: (a) ambiguity concerning the illness, (b) complexity of treatment, (c) lack of or inconsistent information about diagnosis and severity, and (d) unpredictability of prognosis (Mishel, 1988). There is uncertainty surrounding long COVID; research shows that individuals with long COVID report uncertainty surrounding their illness trajectories, with fluctuating periods of good and bad days leading to broader concerns about their future employment and family life (Burton et al., 2022; Kingstone et al., 2020; Ladds et al., 2020).

### Support and Information

Finally, expectations can also be influenced by the support and information given for managing the illness, with a lack of information about the illness (Edwards et al., 2008), and poor signposting to further resources (Swain et al., 2007) leading to poorer outcomes. A lack of signposting to further resources can leave patients unable to find reliable information, making it difficult for them to recognize and monitor their own symptoms and access further advice and support (Edwards et al., 2008). In line with the Uncertainty in Illness Theory, this lack of information may increase the uncertainty surrounding an illness, which may influence the controllability constructs within the Common-Sense model of Self-Regulation, as a lack of information may reduce the perception that an individual has control over their illness (either through their own actions or their treatment plan; Leventhal et al., 2016; Mishel, 1988). Indeed, patients want to be informed of disease-modifying therapies, service entitlement and a treatment plan (Edwards et al., 2008), which, in turn, can promote adherence to suggested treatment and lead to better outcomes (Anestis et al., 2020; Leventhal et al., 1965). However, for long COVID there are difficulties accessing services, with many feeling frustrated that treatments and services are either unavailable, inaccessible, difficult to access, or not being offered (Burton et al., 2022; Ladds et al., 2020).

## The Current Study

The current literature highlights that illness description, symptom uncertainty, and lack of support and information can all contribute to negative expectations of one’s illness, which in turn are associated with poorer health outcomes (Webster et al., 2016). Communication around these three aspects is likely to play a key role in shaping illness expectations and health outcomes associated with long COVID, particularly given the current lack of clarity surrounding the illness. Aside from the physiological factors, the extent to which people experience long COVID symptoms may also be affected by (a) the definition of long COVID (i.e., whether the symptoms are described as ongoing COVID-19 recovery or redefined as a new syndrome; e.g., long COVID); (b) the extent to which the likely prognosis is communicated to be uncertain; (c) the extent to which those experiencing symptoms are given appropriate information and support. To examine the effect of different communication strategies on expectations of long COVID, the current study used an online experiment to manipulate the information given relating to symptom uncertainty (uncertainty emphasized vs. uncertainty not emphasized), illness definition (long COVID vs. ongoing COVID-19 recovery), and amount of support provided (enhanced description of support vs. basic support). Outcomes included expectations about various different aspects of long COVID (symptom severity, symptom duration, quality of life, personal control, treatment control, illness coherence), with expectations being more negative to the extent that expected symptom duration and severity were increased, and expected quality of life, personal control, treatment control, and illness coherence were reduced.

## Primary Hypotheses

1 Illness description: We hypothesized that participants would report more negative expectations (increased symptom severity, increased symptom duration, reduced quality of life, reduced illness coherence, reduced personal control and reduced treatment control) when symptoms were described as long COVID rather than ongoing COVID-19 recovery.
2 Symptom uncertainty: We hypothesized that participants would report more negative expectations (increased symptom severity, increased symptom duration, reduced quality of life, reduced illness coherence, reduced personal control and reduced treatment control) when uncertainty of symptoms was emphasized compared to when uncertainty of symptoms was not emphasized.
3 Efficacy of support: We hypothesized that participants would report more negative expectations (increased symptom severity, increased symptom duration, reduced quality of life, reduced illness coherence, reduced personal control and reduced treatment control) when basic information about available support was described compared to enhanced information about available support.

## Secondary Hypotheses

Based on the literature, we postulated that the condition that would lead to in the most negative illness expectations would include the long COVID description, emphasize symptom uncertainty, and provide basic support (referred to as the hypothesized “worst” condition). We also postulated that the condition that resulted in the most positive illness expectations would include the ongoing COVID-19 recovery description, not emphasize symptom uncertainty, and provide enhanced support (referred to as the hypothesized “best” condition). We hypothesized that participants in the worst condition would report more negative expectations than participants in the best condition. Currently, no research has assessed the combined effect of illness description, symptom uncertainty, and efficacy of support on illness expectations. Therefore, we also explored the interaction between illness description, symptom uncertainty and efficacy of support.

## Method

Ethical approval was obtained from Public Health England’s Research and Ethical Governance Group, participants all gave informed consent before participating. This project followed the Consolidated Standards of Reporting Trials (CONSORT) reporting guideline (Schulz et al., 2010). The CONSORT checklist for each item is included in Supplementary File 1 in the online supplemental materials.

### Transparency and Openness

In this article, we report how we determined our sample size, all data exclusions, all manipulations, and all measures that were included in the study. We follow the Consolidated Standards of Reporting Trials (CONSORT) reporting guideline (Schulz et al., 2010). Data were analyzed using jamovi 1.6.23.0 (The jamovi Project, 2021). The data are available from the authors upon reasonable request. The experiment was preregistered, with the full protocol, on Open Science Frame (https://www.osf.io/7yq56).

### Design

This study was an online survey experiment with a between-subjects design with a 2 (Illness Description: long COVID vs. ongoing COVID-19 recovery) × 2 (Symptom Uncertainty: uncertainty emphasized vs uncertainty not emphasized) × 2 (Efficacy of Support: enhanced support vs basic support) structure. This resulted in eight conditions, with each participant being randomly allocated to one condition. The main outcome measure was expectations regarding long COVID, including expectations of symptom duration, symptom severity, quality of life, personal control, treatment control and illness coherence.

### Participants

Participants were invited to complete the survey if they met the inclusion criteria of being older than 18 years old, residing in the United Kingdom, not having had a confirmed COVID-19 positive test result, and being fluent in English. We excluded those that had a positive COVID-19 test result to avoid results being influenced by participants’ prior personal experiences of COVID-19 or long COVID. Power calculations using G∗Power 3.1 (Faul et al., 2007) required a sample size of 1,014 for a 2 × 2 × 2 design, achieving 95% power to detect a small effect size (*f* = .1). To account for participants failing the attention check question (estimated to be 10%), we aimed to recruit 1,120 participants. We recruited 1,129 participants, with 19 participants removed due to not completing all measures (*n* = 7) or failing the attention check (*n* = 12), leaving a final sample size of 1,110. We recruited participants through Prolific with monetary compensation of £1.25 based on an expected duration of 10 minutes to complete the survey, according to Prolific’s recommended hourly rate. The survey was piloted with 16 participants to check randomization procedures, question coherence, obtain any feedback.

### Measures

#### Scenarios

Participants were presented with a hypothetical two-part scenario, developed by the research team based on information available from the NHS (NHS England and NHS Improvement Coronavirus, 2021), National Institute for Health and Care Excellence (2020a, 2020b, 2020c, 2021), Office for National Statistics (2021) and discussions with GPs. The first part of the scenario, which all participants received, described a positive COVID-19 test result. This part of the scenario was designed to set the scene for the second part of the scenario. The second part of the scenario involved a hypothetical GP appointment that contained information relating to: illness description (long COVID or ongoing COVID-19 recovery); symptom uncertainty (emphasized or not emphasized); and available support (enhanced or basic), which was in line with management and referral guidance (National Institute for Health and Care Excellence, 2021). This resulted in eight variations of the second part of the scenario (see Table 2). Participants were asked to imagine themselves in the given scenarios and answer questions relating to their expectations of the illness, based on the information provided. See Supplementary File 2 in the online supplemental materials for a copy of the scenarios used during the study.[Table tbl2]

#### Outcome Measures

The outcomes for this study were expectations of long COVID, including expected symptom severity, expected symptom duration, expected quality of life, expected personal control (i.e., the extent to which an individual believes they can control their symptoms), expected treatment control (i.e., the extent to which an individual's believe their treatment is effective against their symptoms) and expected illness coherence (i.e., the extent to which an individual understands their illness).

The validated Revised Illness Perception Questionnaire (IPQ-R; Moss-Morris et al., 2002) was used to measure expected symptom severity, expected symptom duration, expected personal control, expected treatment control, and expected illness coherence. The authors of the IPQ-R encourage that the questions be adapted to suit particular illnesses (Moss-Morris et al., 2002); therefore, the questions were adapted from the original present tense to be in the conditional tense, based on expectations. All questions were presented as statements and participants were asked to rate their agreement on a Likert scale from 1 (*Strongly Disagree*) to 5 (*Strongly Agree*).

##### Expected Symptom Severity

We used two adapted subscales from the IPQ-R to capture expected symptom severity: consequences (e.g., “I would expect my illness to be a serious condition”, 6 items, α = .80) and emotional representation (e.g., “I would expect to get depressed when I thought about my illness”, 8 items, α = .85). We added two additional questions to the Emotional Representation subscale to assess perception of expected symptom severity that were adapted from a previous perception scale (Municipal Public Health Service Rotterdam-Rijnmond and National Institute for Public Health and the Environment in the Netherlands, 2015), “having received this diagnosis, I would expect my symptoms to be serious”; “I would be concerned about my symptoms.”

##### Expected Symptom Duration

Expected symptom duration (α = .86) was measured using the “Timeline Acute/Chronic” subscale of the IPQ-R that included six items such as “I would expect my illness to pass quickly.”

##### Expected Symptom Duration in Months

We also measured expected symptom duration in months as a separate outcome variable. Participants were asked an additional question regarding how much longer they would expect to experience their symptoms for with options including less than a month, 1 month, 2–3 months, 4–6 months, 7–9 months, 9–12 months, 12+ months.

##### Expected Personal Control

Personal control (α = .83) assessed participants’ expected control over their symptoms. This was measured using six items adapted questions from the Personal Control subscale of the IPQ-R that included items such as “I would expect to have the power to influence my illness.”

##### Expected Treatment Control

Treatment control (α = .83) assessed participants’ perceptions of efficacy of treatment options. This was measured using 5-items adapted questions from the ‘Treatment Control’ subscale of the IPQ-R such as “I would expect my treatment to be effective in curing my illness.”

##### Expected Illness Coherence

Illness coherence (α = .90) assessed how the illness was understood by participants. This was measured using five items from the Illness Coherence subscale of the IPQ-R such as “I would expect my illness to be a mystery to me.”

##### Expected Quality of Life

An adapted version of the World Health Organization’s Quality of Life questionnaire (World Health Organization, 2012) was used to measure quality of life (α = .81). Four items were presented as statements, with participants asked to rate their agreement such as “I would not expect my future quality of life to be good.”

#### Manipulation Checks

The following manipulation checks were asked to ensure that the conditions were perceived as intended. To assess whether participants considered their symptoms to be associated with long COVID or ongoing COVID-19 recovery, participants were asked: “Considering the information that you have just read, what do you think is the primary cause of your symptoms?” and given the options of (a) ongoing COVID-19 recovery; (b) long COVID; (c) another health condition; (d) do not know. The first two options for this question were randomized to prevent an order effect. To assess participants’ expectations of symptom uncertainty (α = .81), an adapted version of the Timeline Cyclical theme from the IPQ-R (Moss-Morris et al., 2002) was used, whereby the original present tense was changed to be in the conditional tense, based on expectations. To assess the perceived efficacy of support (α = .63), participants were asked to rate their agreement with the following statements: “I would find the support provided by the GP helpful” and “I would know what support was available for my diagnosis.” To check that participants could visualize the scenarios (α = .76), participants were asked to rate their agreement with the following statements “I was able to imagine this situation well” and “I was able to emotionally engage with this situation.” All questions were presented as statements, in a randomized order, and participants were asked to rate their agreement on a Likert scale from 1 (*Strongly Disagree*) to 5 (*Strongly Agree*).

#### Additional Questions

Participants were required to complete an attention check question to check their engagement with the scenarios and the study. Participants were also asked questions relating to their demographic information (age, gender, ethnicity, education, and U.K. region), their existing health conditions, whether they knew someone with long COVID and, if so, what their symptoms were and how long they lasted. These variables were collected to understand if they might influence the participants’ responses to questions on negative expectations.

### Procedure

The data was collected between 14th–16th October 2021. On the 16th October 2021, there had been over 8.5 million positive cases of COVID-19 in the United Kingdom, with an average of 47,082 cases per day. With regards to COVID-19 vaccination, 49.40 million people had received their first dose, 45.36 million people had received their second dose and 3.96 million had received their booster dose (UK Government, 2022). There was also variability with regards to the restrictions in place across the United Kingdom. In Northern Ireland, nightclubs were shut, people had to be sat down in hospitality venues and there was an overall cap of 30 individuals mixing in private dwellings (Office for National Statistics, 2021). Scotland and Wales required face coverings for indoor public settings, and had variations of COVID-19 certification in place for entry to nightclubs and large indoor and outdoor events (Senedd Research, 2022; SPICe Spotlight, 2022).

After consenting to the study, participants were presented with the first scenario asking them to imagine that they had received a positive COVID-19 test result. Participants were then asked to answer questions about expected symptom duration and severity. They were then randomly assigned through Qualtrics to receive one of the eight scenarios describing a long COVID diagnosis. Participants and researchers were blinded to the randomization. After the scenario, participants completed the measures in the following order: visualization check, illness coherence, manipulation checks (symptom uncertainty and efficacy of support), expected symptom severity (consequences and emotional representation), expected symptom duration, expected personal control, expected treatment control, expected quality of life, manipulation check (illness attribution), attention check, and demographics.

### Analytic Strategy

We first used descriptive statistics to describe demographics and then assessed differences in demographics between conditions using χ^2^. Next, we conducted manipulation checks using independent *t*-tests and χ^2^. Finally, we used 2 × 2 × 2 ANOVAs and χ^2^ to assess the impact of assigned conditions on expected symptom severity (consequences and emotional representation), expected symptom duration, expected symptom duration in months, expected quality of life, expected personal control, expected treatment control, and expected illness coherence. We also conducted a series of independent *t*-tests by hypothesized best versus hypothesized worst condition (Scenario 8 vs. Scenario 1) on the outcome measures.

## Results

### Demographics

A breakdown of the demographic characteristics is presented in Table 3. The majority of participants were White (77.9%) and did not know anyone with long COVID (63.1%). Of those who did know someone with long COVID, 74.7% reported that they knew someone with moderate or severe long COVID symptoms, and 43.4% reported that they knew someone whose symptoms were still ongoing. There were no significant differences in demographics or long COVID in friends and family between conditions suggesting that randomization was successful (see the online supplementary materials).[Table tbl3]

### Manipulation Checks

First, we conducted a chi-squared test to examine association between illness description condition (long COVID vs. ongoing COVID-19 recovery) and attribution of illness (long COVID vs. ongoing COVID-19 recovery). The relationship was significant, χ^2^(1) = 60.2, *p* < .001, *V* = .29, with participants more likely to label the attribution as ongoing COVID-19 recovery in the ongoing COVID-19 recovery conditions (76.3%) compared to the long COVID conditions (23.7%). Participants were more likely to label the attribution as long COVID in the long COVID conditions (58.3%) than in the ongoing COVID-19 recovery conditions (41.7%).

We then ran an independent *t* test to assess the effect of the symptom uncertainty manipulation (uncertainty emphasized vs. uncertainty not emphasized) on perceived uncertainty. The results showed participants in the uncertainty emphasized conditions (*M* = 3.68, *SD* = 3.75) reported significantly higher perceived uncertainty than participants in the uncertainty not emphasized conditions (*M* = 3.09, *SD* = 3.00), *t*(698) = 11.7, *p* < .001, *d* = .89.

Last, we ran an independent *t* test to assess the effect of the efficacy of support manipulation (enhanced support vs. basic support) on perceived efficacy of support. The results showed that participants in the enhanced support conditions (*M* = 3.94, *SD*= 4.00) reported significantly greater perceived efficacy of support than participants in the basic support conditions (*M* = 3.82, *SD* = 4.00), *t*(698) = 2.30, *p* = .022, *d* = .17. Overall, the manipulation checks show that the conditions were effective at inducing illness attribution, perceived uncertainty, and perceived efficacy of support, respectively.

### Effect of Condition

Then we ran a series of 2 (Illness Description: long COVID vs. ongoing COVID-19 recovery) × 2 (Symptom Uncertainty: uncertainty emphasized vs. uncertainty not emphasized) × 2 (Efficacy of Support: enhanced support vs. basic support) ANOVAs to test any differences in expectations of symptom severity (consequences and emotional representation), symptom duration, quality of life, personal control, treatment control, and illness coherence between conditions. The results from the ANOVAs are presented in Tables 4 and 5.^1^ We also ran a chi-squared test to assess associations between expected symptom duration in months and conditions.[Table tbl4][Table tbl5]

#### Symptom Severity

The results show no main effect of condition or interacting effect of conditions on expected symptom severity in relation to either consequences or emotional representation.

#### Symptom Duration

The results showed two significant main effects on expected symptom duration: illness description and symptom uncertainty. In terms of illness description, participants in the long COVID conditions reported higher expected symptom duration than participants in the ongoing COVID-19 recovery conditions (*p* < .001, *d* = .23). In regard to symptom uncertainty, participants in the uncertainty emphasized conditions reported higher expected illness duration than participants in the uncertainty not emphasized conditions (*p* < .001, *d* = .34). There was no main effect of efficacy of support or interacting effects.

We also ran three chi squared tests to assess the association between expected symptom duration in months and the three conditions respectively (illness description, symptom uncertainty, efficacy of support). There was a significant association between symptom uncertainty and months of expected symptom duration, χ^2^(5) = 46.7, *p* < .001, *V* = .21. The majority (58.1%) of participants in the uncertainty not emphasized conditions reported expected symptom duration of under 6 months. However, the majority of participants in the uncertainty emphasized conditions (59.3%) expected symptom duration of over 6 months. There was no association between months of expected symptom duration and illness description or efficacy of support.

#### Quality of Life

The results showed no main effect or interacting effects on expected quality of life.

#### Personal Control

The results revealed a main effect of efficacy of support. Participants in the enhanced support conditions reported higher expected personal control than participants in the basic support conditions, *p* = .004, *d* = .17. No other main effects or interacting effects were significant.

#### Treatment Control

The results showed two significant main effects on expected treatment control: symptom uncertainty and efficacy of support. In regard to symptom uncertainty, participants in the uncertainty not emphasized conditions reported higher expected treatment control than participants in the uncertainty emphasized conditions (*p* = .031, *d* = .13). In terms of efficacy of support, participants in the enhanced support conditions reported higher expected treatment control than participants in the basic support conditions (*p* = .037, *d* = .13). The main effect of illness description was not significant and no significant interacting effects were observed.

#### Illness Coherence

The results showed two significant main effects on illness coherence: illness description and symptom uncertainty. In regard to illness description, participants in the ongoing COVID-19 recovery conditions reported significantly higher expected illness coherence than participants in the long COVID conditions, *p* = .029, *d* = .13. Additionally, in terms of symptom uncertainty, participants in the uncertainty not emphasized conditions reported higher expected illness coherence than participants in the uncertainty emphasized conditions, *p* < .001, *d* = .51. The interaction between illness description and efficacy of support had a significant effect on expected illness coherence: Participants in the ongoing COVID-19 recovery and enhanced support condition reported higher expected illness coherence than participants in the long COVID and basic support condition, *p* = .016, *d* = .26.

### Difference Between Expected Best and Worst Condition

We expected to see a difference between the postulated worst condition (long COVID + uncertainty emphasized + basic support; Scenario 1) and the postulated best condition (ongoing COVID-19 recovery + uncertainty not emphasized + enhanced support; Scenario 8). We conducted a series of independent *t*-tests by condition (Scenario 1 vs. Scenario 8) on expected symptom severity (consequences and emotional representation), symptom duration, quality of life, personal control, treatment control, and illness coherence. There were significant differences between Scenario 1 and Scenario 8 in expected symptom duration, treatment control, and illness coherence. Participants in Scenario 8 (ongoing COVID-19 recovery + uncertainty not emphasized + enhanced support) reported shorter expected symptom duration, higher expected treatment control, and higher expected illness coherence than participants in Scenario 1. The results from the *t*-tests are presented in Table 6.[Table tbl6]

## Discussion

We carried out an online experiment to examine the effect of different communication strategies on expectations of long COVID. Specifically, we hypothesized that altering the uncertainty of the illness, the name of the condition, and the level of support provided would affect illness expectations. We found a significant effect of our conditions on four of the six measures of illness expectations (symptom duration, treatment control, personal control, and illness coherence). When the illness was described as long COVID rather than ongoing COVID-19 recovery, participants reported longer expected symptom duration and reduced understanding of their illness (illness coherence), both to a small effect size. When symptom uncertainty was emphasized, participants reported longer expected symptom duration, lower expected efficacy of treatment (treatment control), and less understanding of their illness, to small and medium effect sizes. When information about support was enhanced, compared to basic support information, participants reported higher expected control over their own symptom management (personal control) and higher expected efficacy of treatment, both to a small effect size. Additionally, participants who received enhanced support information, and an illness description of ongoing COVID-19 recovery, reported greater understanding of their illness than those who received basic support information and an illness description of long COVID, to a small effect size.

Our results broadly supported our hypotheses that emphasizing symptom uncertainty, defining symptoms as long COVID, and providing limited information about available support, would increase negative expectations about long COVID. Thus, we found that changing the way in which long COVID symptoms and the support available were described affected negative expectations of illness outcomes. One reason for this is that the term *ongoing COVID-19 recovery* can emphasize hope and optimism for recovery compared to the term *long COVID*. Previous research has indicated that a key component of recovery is hope and therapeutic optimism, which could be facilitated by incorporating recovery into the terminology for long COVID (Craig, 2008; Jones & Evans, 2008; Slade, 2009). Our findings are in line with the extant literature regarding symptom uncertainty and health outcomes, as symptom uncertainty is associated with increased symptom severity, lack of personal control, decline in mental health, and diminished quality of life, among other outcomes in those living with a chronic condition (Wright et al., 2009). The study also provides support for the Common-Sense model of Self-Regulation, which posits that illness description and symptoms help to shape illness perceptions (Leventhal et al., 2016).

However, this study did not find any differences between expected quality of life or expected symptom severity in any of the conditions. One explanation for this could be that the other measures, such as expected symptom duration, illness coherence, and personal and treatment control, may be easier to imagine than one’s expected quality of life. This could be due to the hypothetical nature of the experiment, which may have made it difficult for participants to extrapolate to a real-world context. Additionally, quality of life can be influenced by a myriad of factors, including stress or depression (that often accompany physiological conditions [Pagnini, 2019]), which may also have made it more difficult for participants to accurately imagine their expected quality of life following a long COVID diagnosis.

### Implications and Recommendations

Our findings suggest that the language used to inform people about long COVID, including its symptoms and treatment, can play a role in shaping illness expectations. Information which emphasizes symptom uncertainty, describes symptoms as long COVID, and fails to provide adequate information about the support available, can increase negative expectations regarding the illness. Given the well-established relationship between negative illness expectations and more adverse health outcomes (Pagnini, 2019; Sawyer et al., 2019; Webster et al., 2016), such negative expectations could have an adverse impact on health outcomes for those experiencing symptoms of long COVID.

The findings from this study therefore emphasize the importance of recognizing that the way in which long COVID is communicated can affect illness expectations (and therefore potentially affect health outcomes) and ensuring that communications around long COVID are carefully considered. Specifically, communication about long COVID should provide transparent and factual information about symptoms and available support, while not overemphasizing the uncertainty of symptom severity and duration. While it is important not to set up falsely positive expectations, especially given that data are limited, and the illness is relatively new, individuals should be provided with information on how they can personally facilitate their recovery, as well as where they can access additional support. Our findings also suggest that it may be beneficial to use the term ongoing COVID-19 recovery when referring to ongoing COVID-19 symptoms, though this may be challenging given the prominence of the term long COVID in the narrative around this illness.

### Limitations and Future Research

This study is novel in using an experimental design to assess the effect of different messages about long COVID on expectations of illness outcomes. A limitation of this research is that the study used a design that required participants to imagine themselves in a hypothetical scenario and report their illness expectations based on the information provided. While the results are in line with previous research, including that carried out in real-world contexts, caution should therefore be taken when extrapolating current findings to a real-world context. A related limitation is that the hypothetical nature of the experiment meant that we were unable to measure actual health outcomes. While previous research into other conditions demonstrates the relationship between negative illness expectations and adverse health outcomes (Di Blasi et al., 2001; King & Mishel, 1986; Wright et al., 2009), the area would benefit from further research to explore the relationship between negative expectations and long COVID health outcomes, potentially using a longitudinal design. Also, this study looks at uncertainty regarding the unpredictability of symptoms. However, there are also other types of uncertainty regarding scientific uncertainty that may be particularly pertinent following a long COVID diagnosis (Han et al., 2011). As such, future research could assess the impact of different types of uncertainty on expectations associated with a long COVID diagnosis. A final limitation is that, although the sample was representative of the U.K. population, given that the majority U.K. population are White British, the findings are less generalizable to other ethnicities. This study did not intend to explore any demographic differences, and we found no significant differences in demographic variables between groups, and so it is unlikely that demographic variables affected the outcomes of the study. However, further research is needed to explore the applicability of the current findings to those from other ethnic backgrounds.

### Conclusion

The term long COVID is used to describe symptoms that develop during or after a COVID-19 infection, which continue for more than 4 weeks and cannot be explained by an alternative diagnosis. We assessed the impact of different types of information on illness expectations associated with a hypothetical long COVID diagnosis. We found that describing symptoms as long COVID, emphasizing symptom uncertainty, and reducing the description of available support contributed to more negative illness expectations. In light of the well-established link between negative illness expectations and adverse health outcomes, our findings suggest that communications about long COVID should not emphasize symptom uncertainty and should provide people with information on how they can facilitate their recovery and where they can access additional support. It may also be beneficial to consider using the term ongoing COVID-19 recovery, where possible.

## Supplementary Material

10.1037/hea0001230.supp

## Figures and Tables

**Table 1 tbl1:** Definition for Constructs Within the Common-Sense Model of Self-Regulation

Common-sense model of self-regulation construct	Definition
Identity	A label or name and perceptions of associated symptoms/conditions
Timeline	Perceived or measured rates of onset, duration, and decline
Consequences	Experienced and anticipated physical, cognitive, and social disruption
Causes	Causes of the symptoms (e.g., stress as a cause for heart attacks)
Control	The extent to which you and the prescribed treatment have control of the illness
*Note*. Data from Leventhal et al. (2016).	

**Table 2 tbl2:** Overview of Conditions

Scenario	Condition	*N*	Participants excluded due to attention checks (*n*)	Participants excluded due to incompletion (*n*)
1	Long COVID + Uncertainty emphasized + Basic support condition	137	3	1
2	Long COVID + Uncertainty emphasized + Enhanced support condition	137	3	1
3	Long COVID + Uncertainty not emphasized + Basic support condition	140	1	0
4	Long COVID + Uncertainty not emphasized + Enhanced support condition	140	0	1
5	Ongoing COVID-19 recovery + Uncertainty emphasized + Basic support condition	139	1	1
6	Ongoing COVID-19 recovery + Uncertainty emphasized + Enhanced support condition	138	3	1
7	Ongoing COVID-19 recovery + Uncertainty not emphasized + Basic support condition	139	0	2
8	Ongoing COVID-19 recovery + Uncertainty not emphasized + Enhanced support condition	140	1	0

**Table 3 tbl3:** Participant Demographics

Demographic	*N*	%
Gender		
Woman	562	50.6
Man	538	48.5
Nonbinary	6	0.5
Prefer not to say	4	0.4
Age		
18−24	123	11.1
25−34	226	20.4
35−44	216	19.5
45−54	181	16.3
55−64	233	21.0
65−74	108	9.7
75+	23	2.1
Ethnicity		
Asian	93	8.4
Arab	6	0.5
Black	37	3.3
Hispanic	1	0.1
Mixed	34	3.1
White U.K.	861	77.9
White other	73	6.6
Education		
General Certificate of Secondary Education (GCSE) equivalent or below	166	15.0
A level or equivalent	319	28.7
Undergraduate degree	402	36.2
Postgraduate degree (Masters)	182	16.4
Postgraduate degree (Doctorate)	35	3.2
U.K. region		
NI/Scotland/Wales	154	13.9
England–South	313	28.2
England–London	101	9.1
England–Midlands	263	23.7
England–North	279	25.1
Friend or family with long COVID		
Yes	313	28.2
No	700	63.1
Don’t know	97	8.7
Friend or family long COVID severity		
Mild	52	16.7
Moderate	155	49.7
Severe	78	25.0
Very severe	27	8.7
Friend or family long COVID duration		
Less than a month	10	3.3
1−3 months	47	15.5
3−6 months	59	19.4
6−9 months	31	10.2
9−12 months	18	5.9
12+ months	7	2.3
Still ongoing	132	43.4
Baseline expected severity	*M* = 3.40	*SD* = 0.77
Baseline expected duration	*M* = 2.16	*SD* = 0.58

**Table 4 tbl4:** Means and Standard Deviations for Conditions

Outcome measures: 1 (*strongly disagree*)−5 (*strongly agree*)	Long COVID + Uncertainty emphasized + Basic support condition		Long COVID + Uncertainty emphasized + Enhanced support condition		Long COVID + Uncertainty not emphasized + Basic support condition		Long COVID + Uncertainty not emphasized + Enhanced support condition		Ongoing COVID-19 recovery + Uncertainty emphasized + Basic support condition		Ongoing COVID-19 recovery + Uncertainty emphasized + Enhanced support condition		Ongoing COVID-19 recovery + Uncertainty not emphasized + Basic support condition		Ongoing COVID-19 recovery + Uncertainty not emphasized + Enhanced support condition	
	*M*	*SD*	*M*	*SD*	*M*	*SD*	*M*	*SD*	*M*	*SD*	*M*	*SD*	*M*	*SD*	*M*	*SD*
Symptom severity: Consequences	3.25	0.73	3.26	0.72	3.16	0.76	3.19	0.67	3.13	0.67	3.23	0.65	3.17	0.77	3.24	0.67
Symptom severity: Emotional representation	3.28	0.72	3.31	0.70	3.20	0.74	3.22	0.68	3.18	0.67	3.27	0.64	3.18	0.77	3.23	0.69
Symptom duration	3.01	0.74	3.03	0.72	2.74	0.69	2.80	0.65	2.79	0.70	2.90	0.65	2.70	0.66	2.57	0.66
Quality of life	3.28	0.75	3.27	0.80	3.17	0.75	3.19	0.74	3.17	0.71	3.17	0.71	3.15	0.76	3.18	0.79
Personal control	3.15	0.73	3.33	0.65	3.14	0.71	3.24	0.68	3.07	0.70	3.20	0.65	3.10	0.70	3.18	0.73
Treatment control	3.14	0.77	3.22	0.69	3.20	0.74	3.27	0.72	3.10	0.72	3.20	0.73	3.24	0.70	3.35	0.73
Illness coherence	3.41	0.89	3.34	0.87	3.71	0.79	3.66	0.80	3.95	0.88	3.46	0.78	3.82	0.68	3.95	0.65

**Table 5 tbl5:** ANOVA by Condition on Outcome Variables

Outcome measures: 1 (*strongly disagree*)−5 (*strongly agree*)	Main effect of illness description			Main effect of symptom uncertainty			Main effect of efficacy of support			Interaction effect of illness description and symptom uncertainty			Interaction effect of illness description and efficacy of support			Interaction effect of symptom uncertainty and efficacy of support			Interaction effect of illness description, symptom uncertainty, and efficacy of support		
	*F*(1, 692)	*p*	h_*p*_^2^	*F*(1, 692)	*p*	h_*p*_^2^	*F*(1, 692)	*p*	h_*p*_^2^	*F*(1, 692)	*p*	h_*p*_^2^	*F*(1, 692)	*p*	h_*p*_^2^	*F*(1, 692)	*p*	h_*p*_^2^	*F*(1, 692)	*p*	h_*p*_^2^
Symptom severity: Consequences	0.26	.609	0.00	0.79	0.373	0.00	0.60	0.44	0.00	0.47	.492	0.00	1.09	.296	0.00	0.26	0.607	0.00	1.11	.292	0.00
Symptom severity: Emotional representation	0.53	.466	0.00	2.51	.113	0.00	0.36	0.547	0.00	0.13	.720	0.00	1.44	.230	0.00	0.08	0.783	0.00	1.07	.300	0.00
Symptom duration	6.70	<.001	0.01	31.78	<.001	0.03	0.02	.656	0.00	0.26	.610	0.00	0.32	.574	0.00	1.59	.208	0.00	2.90	.089	0.00
Quality of life	0.58	.446	0.00	2.47	.117	0.00	0.70	.402	0.00	0.19	.661	0.00	0.49	.482	0.00	0.04	.843	0.00	0.29	.592	0.00
Personal control	3.29	.070	0.00	0.31	.578	0.00	8.14	0.00	0.01	0.41	.523	0.00	0.14	.706	0.00	0.63	.429	0.00	0.02	.878	0.00
Treatment control	0.13	.717	0.00	4.65	.031	0.00	4.38	.037	0.00	1.07	.302	0.00	0.11	.737	0.00	0.00	.987	0.00	0.03	.866	0.00
Illness coherence	4.80	.029	0.00	71.44	<.001	0.06	0.65	.420	0.00	3.47	.063	0.00	4.32	.038	0.00	0.00	.987	0.00	0.02	.880	0.00
*Note*. h_*p*_^2^ refers to partial eta squared.																				

**Table 6 tbl6:** t-Test Between Best and Worst Condition

Condition	*MD*	*SE*	*t*	*p*	*df*	Cohen’s *d*
Symptom severity: Consequences	0.02	0.09	0.20	.839	275	0.02
Symptom severity: Emotional representation	0.05	0.09	0.61	.544	275	0.07
Symptom duration	0.03	0.07	5.20	<.001	275	0.63
Quality of life	0.09	0.09	0.99	.324	275	0.12
Personal control	−0.03	0.09	−0.30	.764	275	−0.04
Treatment control	−0.21	0.09	−2.31	.021	275	−0.28
Illness coherence	−0.54	0.09	−5.77	<.001	275	−0.69
*Note*. MD = mean difference. Best condition refers to the condition with ongoing COVID-19 recovery, uncertainty not emphasized and enhanced support. Worst condition refers to the condition with long COVID, uncertainty emphasized and basic.																					

## Footnotes

1 We also ran ANOVAs with gender, age, and contact of long COVID, respectively. We found a main effect of age on illness coherence and an interacting effect of illness description and age on symptom duration and personal control. In terms of gender, we found main effect of gender on emotional representation, symptom duration, personal control, and treatment control and an interacting effect of symptom uncertainty conditions, efficacy of support conditions, and gender on symptom duration. In terms of contact of long COVID, we found a main effect of contact of long COVID on expected symptom duration. See the online supplemental materials for results. Due to limited interacting effects with condition we did not add age, gender, or contact of long COVID as a covariate in the main analyses.

## References

[ref1] Anestis E., Eccles F., Fletcher I., French M., & Simpson J. (2020). Giving and receiving a diagnosis of a progressive neurological condition: A scoping review of doctors’ and patients’ perspectives. Patient Education and Counseling, 103(9), 1709–1723. 10.1016/j.pec.2020.03.023 32299642

[ref2] Broadbent E., Kydd R., Sanders D., & Vanderpyl J. (2008). Unmet needs and treatment seeking in high users of mental health services: Role of illness perceptions. Australian & New Zealand Journal of Psychiatry, 42(2), 147–153. 18197510 10.1080/00048670701787503

[ref3] Burton A., Aughterson H., Fancourt D., & Philip K. E. J. (2022). Factors shaping the mental health and well-being of people experiencing persistent COVID-19 symptoms or ‘long COVID’: Qualitative study. BJPsych Open, 8(2), Article e72. 10.1192/bjo.2022.38 35307048 PMC8987646

[ref4] Clauw D. J., Engel C. C. Jr., Aronowitz R., Jones E., Kipen H. M., Kroenke K., Ratzan S., Sharpe M., & Wessely S. (2003). Unexplained symptoms after terrorism and war: An expert consensus statement. Journal of Occupational and Environmental Medicine, 45(10), 1040–1048. 10.1097/01.jom.0000091693.43121.2f 14534444

[ref5] Copp T., McCaffery K., Azizi L., Doust J., Mol B. W. J., & Jansen J. (2017). Influence of the disease label ‘polycystic ovary syndrome’ on intention to have an ultrasound and psychosocial outcomes: a randomised online study in young women. Human Reproduction, 32(4), 876–884. 10.1093/humrep/dex029 28333180

[ref6] Craig T. K. J. (2008). Recovery: Say what you mean and mean what you say. Journal of Mental Health, 17(2), 125–128. 10.1080/09638230802003800

[ref7] Di Blasi Z., Harkness E., Ernst E., Georgiou A., & Kleijnen J. (2001). Influence of context effects on health outcomes: A systematic review. Lancet, 357(9258), 757–762. 10.1016/s0140-6736(00)04169-6 11253970

[ref8] Edwards R. G., Barlow J. H., & Turner A. P. (2008). Experiences of diagnosis and treatment among people with multiple sclerosis. Journal of Evaluation in Clinical Practice, 14(3), 460–464. 10.1111/j.1365-2753.2007.00902.x 18373567

[ref9] Faul F., Erdfelder E., Lang A.-G., & Buchner A. (2007). G∗Power 3: A flexible statistical power analysis program for the social, behavioral, and biomedical sciences. Behavior Research Methods, 39(2), 175–191. 10.3758/BF03193146 17695343

[ref10] Han P. K. J., Klein W. M. P., & Arora N. K. (2011). Varieties of uncertainty in health care: A conceptual taxonomy. Medical Decision Making: An International Journal of the Society for Medical Decision Making, 31(6), 828–838. 10.1177/0272989x11393976 22067431 PMC3146626

[ref11] Islam M. F., Cotler J., & Jason L. A. (2020). Post-viral fatigue and COVID-19: Lessons from past epidemics. Fatigue: Biomedicine, Health & Behavior, 8(2), 61–69. 10.1080/21641846.2020.1778227

[ref12] The jamovi project. (2021). jamovi (Version 1.6.23.0). https://www.jamovi.org

[ref13] Jones G. H., & Evans H. (2008). Hope, help and recovery. Mental Health Practice, 11(8), 32–37.

[ref14] Karademas E. C. (2006). Self-efficacy, social support and well-being: The mediating role of optimism. Personality and Individual Differences, 40(6), 1281–1290. 10.1016/j.paid.2005.10.019

[ref15] King B., & Mishel M. (1986, April). Uncertainty appraisal and management in chronic illness. Paper presented at the Nineteenth Communicating Nursing Research Conference, Western Society for Research in Nursing, Portland, Oregon.

[ref16] Kingstone T., Taylor A. K., O'Donnell C. A., Atherton H., Blane D. N., & Chew-Graham C. A. (2020). Finding the 'right' GP: A qualitative study of the experiences of people with long-COVID. BJGP Open, 4(5). 10.3399/bjgpopen20X101143 PMC7880173 33051223

[ref17] Ladds E., Rushforth A., Wieringa S., Taylor S., Rayner C., Husain L., & Greenhalgh T. (2020). Persistent symptoms after Covid-19: Qualitative study of 114 “long Covid” patients and draft quality principles for services. BMC Health Services Research, 20(1), Article 1144. 10.1186/s12913-020-06001-y 33342437 PMC7750006

[ref18] Leventhal H., Phillips L. A., & Burns E. (2016). The Common-Sense Model of Self-Regulation (CSM): A dynamic framework for understanding illness self-management. Journal of Behavioral Medicine, 39(6), 935–946. 10.1007/s10865-016-9782-2 27515801

[ref19] Leventhal H., Singer R., & Jones S. (1965). Effects of fear and specificity of recommendation upon attitudes and behavior. Journal of Personality and Social Psychology, 2(1), 20–29. 10.1037/h0022089 14313839

[ref20] McCarthy S. C., Lyons A. C., Weinman J., Talbot R., & Purnell D. (2003). Do expectations influence recovery from oral surgery? An illness representation approach. Psychology & Health, 18(1), 109–126. 10.1080/0887044031000080674

[ref21] Mishel M. H. (1988). Uncertainty in illness. Image: The Journal of Nursing Scholarship, 20(4), 225–232. 10.1111/j.1547-5069.1988.tb00082.x 3203947

[ref22] Moss-Morris R., Petrie K. J., & Weinman J. (1996). Functioning in chronic fatigue syndrome: Do illness perceptions play a regulatory role? British Journal of Health Psychology, 1(1), 15–25. 10.1111/j.2044-8287.1996.tb00488.x

[ref23] Moss-Morris R., Weinman J., Petrie K., Horne R., Cameron L., & Buick D. (2002). The revised Illness Perception Questionnaire (IPQ-R). Psychology & Health, 17(1), 1–16. 10.1080/08870440290001494

[ref24] Municipal Public Health Service Rotterdam-Rijnmond, and National Institute for Public Health and the Environment in the Netherlands. (2015). Standard questionnaire on risk perception of an infectious disease outbreak. Retrieved 20 May, 2021, from http://ecomeu.info/wp-content/uploads/2015/11/Standard-questionnaire-risk-perception-ECOM-november-2015.pdf

[ref25] National Institute for Health and Care Excellence. (2020a). Assessing people with new or ongoing symptoms after acute COVID-19. https://www.nice.org.uk/guidance/ng188/chapter/2-Assessing-people-with-new-or-ongoing-symptoms-after-acute-COVID-19

[ref26] National Institute for Health and Care Excellence. (2020b). Identifying people with ongoing symptomatic COVID-19 or post-COVID-19 syndrome. https://www.nice.org.uk/guidance/ng188/chapter/1-Identifying-people-with-ongoing-symptomatic-COVID-19-or-post-COVID-19-syndrome

[ref27] National Institute for Health and Care Excellence. (2020c). Management. https://www.nice.org.uk/guidance/ng188/chapter/management#self-management-and-supported-self-management

[ref28] National Institute for Health and Care Excellence. (2021). Scenario: Managing long-term effects. https://cks.nice.org.uk/topics/coronavirus-covid-19/management/managing-long-term-effects/

[ref29] NHS England and NHS Improvement Coronavirus. (2021). Post-COVID syndrome (Long COVID). https://www.england.nhs.uk/coronavirus/post-covid-syndrome-long-covid/

[ref30] Office for National Statistics. (2021). Prevalence of ongoing symptoms following coronavirus (COVID-19) infection in the U.K.: 4 November 2021. https://www.ons.gov.uk/peoplepopulationandcommunity/healthandsocialcare/conditionsanddiseases/bulletins/prevalenceofongoingsymptomsfollowingcoronaviruscovid19infectionintheuk/4november2021

[ref31] Pagnini F. (2019). The potential role of illness expectations in the progression of medical diseases. BMC Psychology, 7(1), Article 70. 10.1186/s40359-019-0346-4 31703607 PMC6839057

[ref32] Park C., Pagnini F., & Langer E. (2020). Glucose metabolism responds to perceived sugar intake more than actual sugar intake. Scientific Reports, 10(1), Article 15633. 10.1038/s41598-020-72501-w 32973226 PMC7515886

[ref33] Park C., Pagnini F., Reece A., Phillips D., & Langer E. (2016). Blood sugar level follows perceived time rather than actual time in people with type 2 diabetes. Proceedings of the National Academy of Sciences, 113(29), 8168–8170. 10.1073/pnas.1603444113 PMC4961154 27382161

[ref34] Pennebaker J. (2012). The psychology of physical symptoms. Springer Science & Business Media. 10.1007/978-1-4613-8196-9

[ref35] Pennebaker J., & Skelton A. (1978). Psychological parameters of physical symptoms. Personality and Social Psychology Bulletin, 4(4), 524–530. 10.1177/014616727800400405

[ref36] Petrie K. J., MacKrill K., Derksen C., & Dalbeth N. (2018). An illness by any other name: The effect of renaming gout on illness and treatment perceptions. Health Psychology, 37(1), 37–41. 10.1037/hea0000548 28836797

[ref37] Petrie K. J., & Weinman J. (2006). Why illness perceptions matter. Clinical Medicine, 6(6), 536–539. 10.7861/clinmedicine.6-6-536 17228551 PMC4952762

[ref38] Poenaru S., Abdallah S. J., Corrales-Medina V., & Cowan J. (2021). COVID-19 and post-infectious myalgic encephalomyelitis/chronic fatigue syndrome: A narrative review. Therapeutic Advances in Infectious Disease, 8. 10.1177/20499361211009385 PMC8060761 33959278

[ref39] Sawyer A. T., Harris S. L., & Koenig H. G. (2019). Illness perception and high readmission health outcomes. Health Psychology Open, 6(1). 10.1177/2055102919844504 PMC6482662 31041109

[ref40] Scherer L. D., Zikmund-Fisher B. J., Fagerlin A., & Tarini B. A. (2013). Influence of “GERD” label on parents' decision to medicate infants. Pediatrics, 131(5), 839–845. 10.1542/peds.2012-3070 23545371 PMC3639462

[ref41] Schulz K. F., Altman D. G., Moher D., & the CONSORT Group. (2010). CONSORT 2010 statement: Updated guidelines for reporting parallel group randomised trials. BMJ, 340, c332. 10.1136/bmj.c332 20332509 PMC2844940

[ref42] Senedd Research. (2022). Coronavirus timeline: The response in Wales. Retrieved 4 April, 2022, from https://research.senedd.wales/research-articles/coronavirus-timeline-the-response-in-wales/

[ref43] Slade M. (2009). Personal recovery and mental Illness: A guide for mental health professionals. Cambridge University Press.

[ref44] SPICe Spotlight. (2022). Timeline of coronavirus (COVID-19) in Scotland. Retrieved 4 April, 2022, from https://spice-spotlight.scot/2022/04/01/timeline-of-coronavirus-covid-19-in-scotland/

[ref45] Swain D., Ellins J., Coulter A., Heron P., Howell E., Magee H., Cairncross L., Chisholm A., & Rasul F. (2007). Accessing information about health and social care services. Picker Institute Europe. https://www.academia.edu/22582691/Accessing_information_about_health_and_social_care_services

[ref46] UK Government. (2022). Coronavirus (COVID-19) in the UK. Retrieved 4 April, 2022, from https://coronavirus.data.gov.uk/details/vaccinations

[ref47] Webster R. K., Weinman J., & Rubin G. J. (2016). A systematic review of factors that contribute to nocebo effects. Health Psychology, 35(12), 1334–1355. 10.1037/hea0000416 27657801

[ref48] World Health Organization. (2012). WHOQOL: Measuring quality of life. https://www.who.int/tools/whoqol

[ref49] Wright L. J., Afari N., & Zautra A. (2009). The illness uncertainty concept: A review. Current Pain and Headache Reports, 13(2), 133–138. 10.1007/s11916-009-0023-z 19272279

