# Supplementary material for: The Effects of Messaging on Long COVID Expectations: An Online Experiment
Source: Health Psychol. 2022 Sep 15;41(11):853–63. doi: 10.1037/hea0001230 (PMC9575348; doi:10.1037/hea0001230)
Supplement: Supplementary file 1 [file HEA-2022-3748_Supplemental_Materials.docx]

**The Effects of Messaging on Long COVID Expectations: An Online Experiment**

***Supplementary Files***

***Health Psychology Journal***

Jaskiran Kaur Bhogal*^1^, Freya Mills*^1^, Amelia Dennis, Cristina Spoiala^1^, Joanna Milward^1^, Sidra Saeed^2^, Leah Ffion Jones^1^, Dale Weston^1^, Holly Carter^1^

* Joint first authors, these authors contributed equally to this work

^1^ Behavioural Science and Insights Unit, UKHSA

^2^ Data, Analytics and Surveillance, UKHSA

**Supplementary File 1: Patient and Public Involvement**

We carried out stakeholder involvement in the form of discussions with GPs (*n*=5) to gain an insight into the structure of a typical GP consultation, particularly in relation to cases of suspected Long COVID. We did not carry out patient and public involvement.

**Supplementary File 2: Scenarios**

***Scenario 1:***

A couple of months after testing positive for COVID-19, you are feeling unwell. You make an appointment to see your GP, where you describe some of the symptoms you are experiencing. You describe symptoms including fatigue (extreme tiredness), shortness of breath, coughing, not being able to think straight (brain fog), chest pain and joint and muscle pain.     

***Scenario 2:***

i. Long COVID + Emphasising Uncertainty + No Enhanced Signposting of Information

Your GP explains that the symptoms you are describing may be due to Long COVID. Your GP explains that at the moment, it is uncertain how long your symptoms will last for or how severe they might be. They also tell you that your symptoms may be unpredictable and that they may affect you in different ways at different times. They explain that your symptoms may get better and then come back and that they may also change over time. They provide you with a leaflet with further information about Long COVID and symptom management.

ii. Long COVID + Emphasising Uncertainty + Enhanced Signposting of Information

Your GP explains that the symptoms you are describing may be due to Long COVID. Your GP explains that at the moment, it is uncertain how long your symptoms will last for or how severe they might be. They also tell you that your symptoms may be unpredictable and that they may affect you in different ways at different times. They explain that your symptoms may get better and then come back and that they may also change over time. They discuss your symptoms with you and provide you with a leaflet containing further information about Long COVID. This includes information on symptoms that you might experience, as well as information about what you can do to manage your symptoms at home. Your GP explains that they will work with you to help you understand and manage symptoms, and that there are support groups and online forums that you can go to for further advice and to help assist your recovery.

iii. Long COVID + Not Emphasising Uncertainty + No Enhanced Signposting of Information

Your GP explains that the symptoms you are describing may be due to Long COVID. They explain that most people will make a full recovery quite quickly, but for some people symptoms can last longer.  They provide you with a leaflet with further information about Long COVID and symptom management.

iv. Long COVID + Not Emphasising Uncertainty + Enhanced Signposting of Information

Your GP explains that the symptoms you are describing may be due to Long COVID. They explain that most people will make a full recovery quite quickly, but for some people symptoms can last longer.  They discuss your symptoms with you and provide you with a leaflet containing further information about Long COVID. This includes information on symptoms that you might experience, as well as information about what you can do to manage your symptoms at home. Your GP explains that they will work with you to help you understand and manage symptoms, and that there are support groups and online forums that you can go to for further advice and to help assist your recovery.

v. Ongoing COVID-19 Recovery + Emphasising Uncertainty + No Enhanced Signposting of Information

Your GP explains that the symptoms you are describing may be due to ongoing COVID-19 recovery. Your GP explains that at the moment, it is uncertain how long your symptoms will last for or how severe they might be. They also tell you that your symptoms may be unpredictable and that they may affect you in different ways at different times. They explain that your symptoms may get better and then come back and that they may also change over time. They provide you with a leaflet with further information about ongoing COVID-19 recovery and symptom management.

vi. Ongoing COVID-19 Recovery + Emphasising Uncertainty + Enhanced Efficacy of Signposting of Information

Your GP explains that the symptoms you are describing may be due to ongoing COVID-19 recovery. Your GP explains that at the moment, it is uncertain how long your symptoms will last for or how severe they might be. They also tell you that your symptoms may be unpredictable and that they may affect you in different ways at different times. They explain that your symptoms may get better and then come back and that they may also change over time. They discuss your symptoms with you and provide you with a leaflet containing further information about ongoing COVID-19 recovery. This includes information on symptoms that you might experience, as well as information about what you can do to manage your symptoms at home. Your GP explains that they will work with you to help you understand and manage symptoms, and that there are support groups and online forums that you can go to for further advice and to help assist your recovery.

vii. Ongoing COVID-19 Recovery + Not Emphasising Uncertainty + No Enhanced Signposting of Information

Your GP explains that the symptoms you are describing may be due to ongoing COVID-19 recovery. They explain that most people will make a full recovery quite quickly, but for some people symptoms can last longer. They provide you with a leaflet with further information about ongoing COVID-19 recovery and symptom management.

viii. Ongoing COVID-19 Recovery + Not Emphasising Uncertainty + Enhanced Signposting of Information

Your GP explains that the symptoms you are describing may be due to ongoing COVID-19 recovery. They explain that most people will make a full recovery quite quickly, but for some people symptoms can last longer.   They discuss your symptoms with you and provide you with a leaflet containing further information about ongoing COVID-19 recovery. This includes information on symptoms that you might experience, as well as information about what you can do to manage your symptoms at home. Your GP explains that they will work with you to help you understand and manage symptoms, and that there are support groups and online forums that you can go to for further advice and to help assist your recovery.

**Supplementary File 3: Further Analysis**

**Demographic Differences by Condition**

We ran a series of Chi-Squared association tests and ANOVA by condition on gender, age, ethnicity, education, UK region, friends or family with Long COVID, friend or family Long COVID severity, friends or family Long COVID duration, baseline expected severity and baseline expected duration. The results (see Supplementary Table 1) showed no significant differences by condition on any demographics suggesting randomisation was successful.

**Table 1**

*Demographic differences by condition*

|  | Long COVID + Uncertainty Emphasised + Basic Support Condition | | Long COVID + Uncertainty Emphasised + Enhanced Support Condition | | Long COVID + Uncertainty Not Emphasised + Basic Support Condition | | Long COVID + Uncertainty Not Emphasised + Enhanced Support Condition | | Ongoing COVID-19 Recovery + Uncertainty Emphasised + Basic Support Condition | | Ongoing COVID-19 Recovery + Uncertainty Emphasised + Enhanced Support Condition | | Ongoing COVID-19 Recovery + Uncertainty Not Emphasised + Basic Support Condition | | Ongoing COVID-19 Recovery + Uncertainty Not Emphasised + Enhanced Support Condition | | F/X^2^ | p |
| --- | --- | --- | --- | --- | --- | --- | --- | --- | --- | --- | --- | --- | --- | --- | --- | --- | --- | --- |
|  | n | % | n | % | n | % | n | % | n | % | n | % | n | % | n | % |  |  |
| **Gender** |  |  |  |  |  |  |  |  |  |  |  |  |  |  |  |  | 16.4 | .745 |
| Woman | 70 | 51.1 | 61 | 44.5 | 67 | 47.9 | 67 | 47.9 | 61 | 43.9 | 70 | 50.7 | 72 | 51.8 | 70 | 50.0 |  |  |
| Man | 65 | 47.4 | 76 | 55.5 | 71 | 50.7 | 72 | 41.4 | 77 | 55.4 | 66 | 47.8 | 67 | 48.2 | 68 | 48.6 |  |  |
| Non-Binary | 1 | 0.7 | 0 | 0.0 | 0 | 0.0 | 1 | 0.7 | 1 | 0.7 | 1 | 0.7 | 0 | 0.0 | 2 | 1.4 |  |  |
| Prefer not to say | 1 | 0.7 | 0 | 0.0 | 2 | 1.4 | 0 | 0.0 | 0 | 0.0 | 1 | 0.7 | 0 | 0.0 | 0 | 0.0 |  |  |
| **Age** |  |  |  |  |  |  |  |  |  |  |  |  |  |  |  |  | 46.1 | .308 |
| 18-24 | 14 | 10.2 | 17 | 12.4 | 9 | 6.4 | 12 | 8.6 | 18 | 12.9 | 22 | 15.9 | 15 | 10.8 | 16 | 11.4 |  |  |
| 25-34 | 21 | 15.3 | 33 | 24.1 | 30 | 21.4 | 29 | 20.7 | 27 | 19.4 | 27 | 19.6 | 35 | 25.2 | 24 | 17.1 |  |  |
| 35-44 | 25 | 18.2 | 21 | 15.3 | 34 | 24.3 | 29 | 20.7 | 32 | 23.0 | 24 | 17.4 | 25 | 18.0 | 26 | 18.6 |  |  |
| 45-54 | 31 | 22.6 | 16 | 11.7 | 20 | 14.3 | 25 | 17.9 | 25 | 18.0 | 22 | 15.9 | 21 | 15.1 | 21 | 15.0 |  |  |
| 55-64 | 25 | 18.2 | 29 | 21.2 | 31 | 22.1 | 21 | 15.0 | 30 | 21.6 | 29 | 21.0 | 30 | 21.6 | 38 | 27.1 |  |  |
| 65-74 | 16 | 11.7 | 16 | 11.7 | 15 | 10.7 | 19 | 13.6 | 6 | 4.3 | 12 | 8.7 | 10 | 7.2 | 14 | 10.0 |  |  |
| 75+ | 5 | 3.6 | 5 | 3.6 | 1 | 0.7 | 5 | 3.6 | 1 | 0.7 | 2 | 1.4 | 3 | 2.2 | 1 | 0.7 |  |  |
| **Ethnicity** |  |  |  |  |  |  |  |  |  |  |  |  |  |  |  |  | 41.7 | .482 |
| Asian | 12 | 8.8 | 15 | 11.0 | 12 | 8.6 | 8 | 5.7 | 14 | 10.1 | 13 | 9.5 | 7 | 5.1 | 12 | 8.6 |  |  |
| Arab | 0 | 0.0 | 1 | 0.7 | 0 | 0.0 | 1 | 0.7 | 0 | 0.0 | 1 | 0.7 | 1 | 0.7 | 2 | 1.4 |  |  |
| Black | 4 | 2.9 | 4 | 2.9 | 8 | 5.8 | 6 | 4.3 | 3 | 2.2 | 3 | 2.2 | 1 | 0.7 | 8 | 5.7 |  |  |
| Hispanic | 0 | 0.0 | 0 | 0.0 | 0 | 0.0 | 0 | 0.0 | 0 | 0.0 | 0 | 0.0 | 1 | 0.7 | 0 | 0.0 |  |  |
| Mixed | 4 | 2.9 | 3 | 2.2 | 1 | 0.7 | 4 | 2.9 | 6 | 4.3 | 5 | 3.6 | 8 | 5.8 | 3 | 2.1 |  |  |
| White UK | 112 | 82.4 | 106 | 77.9 | 112 | 80.6 | 108 | 77.1 | 103 | 74.1 | 105 | 76.6 | 110 | 79.7 | 105 | 75.0 |  |  |
| White other | 4 | 2.9 | 7 | 5.1 | 6 | 4.3 | 13 | 9.3 | 13 | 9.4 | 10 | 7.3 | 10 | 7.2 | 10 | 7.1 |  |  |
| **Education** |  |  |  |  |  |  |  |  |  |  |  |  |  |  |  |  | 42.6 | .177 |
| GCSE equivalent or below | 27 | 19.7 | 21 | 15.3 | 16 | 11.4 | 24 | 17.1 | 22 | 15.8 | 18 | 13.0 | 24 | 17.3 | 14 | 10.0 |  |  |
| A level or equivalent | 36 | 26.3 | 33 | 24.1 | 54 | 38.6 | 33 | 23.6 | 42 | 30.2 | 42 | 30.4 | 34 | 24.5 | 45 | 36.2 |  |  |
| Undergraduate degree | 48 | 35.9 | 46 | 34.3 | 50 | 35.7 | 52 | 37.1 | 50 | 36.0 | 43 | 31.2 | 53 | 38.1 | 59 | 42.1 |  |  |
| Postgraduate degree (Masters) | 22 | 16.1 | 29 | 21.2 | 17 | 12.1 | 20 | 14.3 | 20 | 14.4 | 30 | 21.7 | 25 | 18.0 | 19 | 13.6 |  |  |
| Postgraduate degree (Doctorate) | 3 | 2.2 | 7 | 5.1 | 2 | 1.4 | 10 | 7.1 | 4 | 2.9 | 3 | 2.2 | 3 | 2.2 | 3 | 2.2 |  |  |
| **UK Region** |  |  |  |  |  |  |  |  |  |  |  |  |  |  |  |  | 23.9 | .684 |
| NI/Scotland/Wales | 14 | 10.2 | 13 | 9.5 | 20 | 14.3 | 30 | 21.4 | 19 | 13.7 | 23 | 16.7 | 17 | 12.2 | 18 | 12.9 |  |  |
| England - South | 44 | 32.1 | 33 | 24.1 | 41 | 29.3 | 37 | 26.4 | 38 | 27.3 | 38 | 27.5 | 43 | 30.9 | 39 | 27.9 |  |  |
| England – London | 12 | 8.8 | 10 | 7.3 | 17 | 12.1 | 11 | 7.9 | 12 | 8.6 | 9 | 6.5 | 13 | 9.4 | 17 | 12.1 |  |  |
| England – Midlands | 33 | 24.1 | 39 | 28.5 | 29 | 20.7 | 35 | 25.0 | 33 | 23.7 | 34 | 24.6 | 28 | 20.1 | 32 | 22.9 |  |  |
| England - North | 33 | 24.1 | 39 | 28.5 | 29 | 20.7 | 35 | 20.0 | 33 | 23.7 | 34 | 24.6 | 28 | 20.1 | 32 | 22.9 |  |  |
| **Friend or Family with Long COVID** |  |  |  |  |  |  |  |  |  |  |  |  |  |  |  |  | 15.0 | .378 |
| Yes | 32 | 23.4 | 36 | 26.3 | 41 | 29.3 | 33 | 23.6 | 45 | 32.4 | 35 | 25.4 | 44 | 31.7 | 47 | 33.6 |  |  |
| No | 93 | 67.9 | 90 | 65.7 | 84 | 90.0 | 94 | 67.1 | 81 | 58.3 | 85 | 61.6 | 88 | 63.3 | 85 | 60.7 |  |  |
| Don’t Know | 12 | 8.8 | 11 | 8.0 | 15 | 10.7 | 13 | 9.3 | 13 | 9.4 | 18 | 13.0 | 7 | 5.0 | 8 | 5.7 |  |  |
| **Friend or Family Long COVID Severity** |  |  |  |  |  |  |  |  |  |  |  |  |  |  |  |  | 18.3 | .629 |
| Mild | 6 | 18.8 | 10 | 27.8 | 5 | 12.5 | 8 | 24.2 | 4 | 8.9 | 9 | 25.7 | 4 | 9.1 | 6 | 12.8 |  |  |
| Moderate | 17 | 53.1 | 13 | 36.1 | 22 | 55.0 | 13 | 39.4 | 26 | 57.8 | 17 | 48.6 | 23 | 52.3 | 24 | 51.1 |  |  |
| Severe | 8 | 25.0 | 9 | 25.0 | 9 | 22.5 | 10 | 30.3 | 10 | 22.2 | 5 | 14.3 | 14 | 31.8 | 13 | 27.7 |  |  |
| Very Severe | 1 | 3.1 | 4 | 11.1 | 4 | 10.0 | 2 | 6.1 | 5 | 11.1 | 4 | 11.4 | 3 | 6.8 | 4 | 8.5 |  |  |
| **Friend or Family Long COVID Duration** |  |  |  |  |  |  |  |  |  |  |  |  |  |  |  |  | 26.6 | .969 |
| Less than a month | 1 | 3.1 | 0 | 0.0 | 2 | 5.3 | 1 | 3.1 | 3 | 6.7 | 2 | 5.9 | 0 | 0.0 | 1 | 2.2 |  |  |
| 1-3 months | 4 | 12.5 | 3 | 8.8 | 8 | 21.1 | 5 | 15.6 | 9 | 20.0 | 3 | 8.8 | 6 | 13.6 | 9 | 20.0 |  |  |
| 3-6 months | 5 | 15.6 | 8 | 23.5 | 6 | 15.8 | 7 | 21.9 | 9 | 20.0 | 6 | 17.6 | 8 | 18.2 | 10 | 22.2 |  |  |
| 6-9 months | 4 | 12.5 | 3 | 8.8 | 3 | 7.9 | 2 | 6.3 | 7 | 15.6 | 3 | 8.8 | 7 | 15.9 | 2 | 4.4 |  |  |
| 9-12 months | 2 | 6.3 | 2 | 5.9 | 2 | 5.3 | 2 | 6.3 | 3 | 6.7 | 2 | 5.9 | 2 | 4.5 | 3 | 6.7 |  |  |
| 12+ months | 1 | 3.1 | 0 | 0.0 | 0 | 0.0 | 1 | 3.1 | 1 | 2.2 | 0 | 0.0 | 1 | 2.3 | 3 | 6.7 |  |  |
| Still ongoing | 15 | 46.9 | 18 | 52.9 | 17 | 44.7 | 14 | 43.8 | 13 | 28.9 | 18 | 52.9 | 20 | 45.5 | 17 | 37.8 |  |  |
| **Baseline Expected Severity** | 3.42 | 0.73 | 3.42 | 0.77 | 3.31 | 0.71 | 3.40 | 0.74 | 3.37 | 0.86 | 3.44 | 0.75 | 3.38 | 0.78 | 3.45 | 0.83 | 0.46 | .867 |
| **Baseline Expected Duration** | 2.18 | 0.64 | 2.18 | 0.60 | 2.16 | 0.54 | 2.17 | 0.57 | 2.10 | 0.60 | 2.16 | 0.52 | 2.19 | 0.58 | 2.15 | 0.56 | 0.33 | .942 |

**The Effect of Contact of Long COVID**

To assess the influence of age, we ran a series 2 (illness description: Long COVID vs. ongoing COVID-19 recovery) X 2 (uncertainty: uncertainty emphasised vs. uncertainty not emphasised) X 2 (efficacy of support: enhanced support vs. basic support) X 2 (contact of Long COVID: yes; no) ANOVAs on symptom severity (consequences and emotional representation), symptom duration, quality of life, personal control, treatment control, and illness coherence. All results for these analyses are presented in Supplementary Table 2. We found a main effect of contact of Long COVID on symptom duration and quality of life. People who knew someone with Long COVID reported higher expected symptom duration (p<.001, d=0.33) and quality of life (p=.043, d=0.14) than those who did not know anyone with Long COIVD. Although we did find a significant interaction of illness description, uncertainty, efficacy of support, and contact of Long COVID on quality of life, post hoc tests for both of these results showed non-significant differences.

**Table 2**

*The main effect and interacting effects of contact of Long COVID*

|  | Main Effect of Contact of Long COVID | | | Interaction Effect of Illness Description and Contact of Long COVID | | | Interaction Effect of and Illness Uncertainty and Contact of Long COVID | | | Interaction Effect of Efficacy of Support and Contact of Long COVID | | | Interaction Effect of Illness Description, Illness Uncertainty, and Contact of Long COVID | | | Interaction Effect of Illness Description, Efficacy of Support, and Contact of Long COVID | | | Interaction Effect of Illness Uncertainty, Efficacy of Support, and Contact of Long COVID | | | Interaction Effect of Illness Description, Illness Uncertainty, Efficacy of Support, and Contact of Long COVID | | |
| --- | --- | --- | --- | --- | --- | --- | --- | --- | --- | --- | --- | --- | --- | --- | --- | --- | --- | --- | --- | --- | --- | --- | --- | --- |
|  | *F*(1, 692) | *p* | η_p_^2^ | *F*(1, 692) | *p* | η_p_^2^ | *F*(1, 692) | *p* | η_p_^2^ | *F*(1, 692) | *p* | η_p_^2^ | *F*(1, 692) | *p* | η_p_^2^ | *F*(1, 692) | *p* | η_p_^2^ | *F*(1, 692) | *p* | η_p_^2^ | *F*(1, 692) | *p* | η_p_^2^ |
| Symptom Severity: Consequences | 2.24 | .135 | 0.00 | 0.24 | .623 | 0.00 | 0.80 | .371 | 0.00 | 0.03 | .861 | 0.00 | 0.00 | .967 | 0.00 | 0.00 | .995 | 0.00 | 1.47 | .225 | 0.01 | 3.49 | .062 | 0.00 |
| Symptom Severity: Emotional Representation | 3.29 | .070 | 0.00 | 0.27 | .604 | 0.00 | 1.48 | .224 | 0.00 | 0.60 | .438 | 0.00 | 0.00 | .981 | 0.00 | 0.34 | .558 | 0.00 | 0.97 | .324 | 0.00 | 3.26 | .071 | 0.00 |
| Symptom Duration | 23.56 | <.001 | 0.02 | 0.78 | .377 | 0.00 | 0.23 | .635 | 0.00 | 1.87 | .172 | 0.00 | 1.11 | .293 | 0.00 | 1.83 | .176 | 0.00 | 0.51 | .474 | 0.00 | 0.13 | .714 | 0.00 |
| Quality of life | 4.12 | .043 | 0.00 | 1.10 | .293 | 0.00 | 3.22 | .073 | 0.00 | 0.89 | .345 | 0.00 | 0.82 | .365 | 0.00 | 0.75 | .388 | 0.00 | 0.44 | .507 | 0.00 | 4.36 | .037 | 0.04 |
| Personal Control | 0.47 | .494 | 0.00 | 0.04 | .851 | 0.00 | 0.93 | .334 | 0.00 | 0.10 | .755 | 0.00 | 0.13 | .722 | 0.00 | 0.01 | .913 | 0.00 | 0.46 | .498 | 0.00 | 1.39 | .239 | 0.00 |
| Treatment Control | 1.13 | .288 | 0.00 | 0.12 | .732 | 0.00 | 2.55 | .111 | 0.00 | 2.09 | .149 | 0.00 | 0.04 | .849 | 0.00 | 0.00 | .994 | 0.00 | 0.49 | .486 | 0.00 | 3.75 | .053 | 0.00 |
| Illness Coherence | 1.03 | .310 | 0.00 | 0.23 | .630 | 0.00 | 0.39 | .535 | 0.00 | 3.59 | .058 | 0.00 | 0.05 | .816 | 0.00 | 0.26 | .614 | 0.00 | 1.59 | .207 | 0.00 | 1.02 | .312 | 0.00 |

**The Effect of Demographics**

To assess the influence of age, we ran a series 2 (illness description: Long COVID vs. ongoing COVID-19 recovery) X 2 (uncertainty: uncertainty emphasised vs. uncertainty not emphasised) X 2 (efficacy of support: enhanced support vs. basic support) X 7 (age: 18-24; 25-34; 35-44; 45-54; 55-64; 65-74; 75+) ANOVAs on symptom severity (consequences and emotional representation), symptom duration, quality of life, personal control, treatment control, and illness coherence. All results for these analyses are presented in Supplementary Table 3. We found a main effect of age on illness coherence. Illness coherence was lower in 25-34 year olds compared to 55-64 (p<.001, d=-0.50) and 65-74 year olds (p<.001, d=-0.54). Second, illness coherence was lower in 35-44 year olds compared to 55-64 (p=.003, d=-0.36) and 65-74 year olds (p=.018, d=-0.40). We also found an interaction between illness description and age on symptom duration and personal control. In terms of symptom duration, 18-24 year olds in the Long COVID condition reported higher symptom duration than 25-34 year olds in the ongoing COVID-19 recovery condition (p=.009, d=-0.59) and 35-44 year olds in the ongoing COVID-19 recovery condition (p=.029, d=-0.55). In terms of personal control, we found 75+ year olds in the Long COVID condition reported less personal control than 65-74 year olds in the ongoing COVID-19 recovery condition (p=.018, d=1.30). Although we did find a significant interaction of uncertainty condition and age on quality of life and personal control, post hoc tests for both of these results showed non-significant differences.

**Supplementary Table 3**

*The main effect and interacting effects of age*

|  | Main Effect of Age | | | Interaction Effect of Illness Description and Age | | | Interaction Effect of and Illness Uncertainty and Age | | | Interaction Effect of Efficacy of Support and Age | | | Interaction Effect of Illness Description, Illness Uncertainty, and Age | | | Interaction Effect of Illness Description, Efficacy of Support, and Age | | | Interaction Effect of Illness Uncertainty, Efficacy of Support, and Age | | | Interaction Effect of Illness Description, Illness Uncertainty, Efficacy of Support, and Age | | |
| --- | --- | --- | --- | --- | --- | --- | --- | --- | --- | --- | --- | --- | --- | --- | --- | --- | --- | --- | --- | --- | --- | --- | --- | --- |
|  | *F*(1, 692) | *p* | η_p_^2^ | *F*(1, 692) | *p* | η_p_^2^ | *F*(1, 692) | *p* | η_p_^2^ | *F*(1, 692) | *p* | η_p_^2^ | *F*(1, 692) | *p* | η_p_^2^ | *F*(1, 692) | *p* | η_p_^2^ | *F*(1, 692) | *p* | η_p_^2^ | *F*(1, 692) | *p* | η_p_^2^ |
| Symptom Severity: Consequences | 1.27 | .267 | 0.01 | 0.59 | .740 | 0.00 | 2.07 | .054 | 0.01 | 0.74 | .620 | 0.00 | 1.08 | .373 | 0.01 | 0.73 | .622 | 0.00 | 0.23 | .966 | 0.00 | 0.39 | .883 | 0.00 |
| Symptom Severity: Emotional Representation | 0.99 | .431 | 0.01 | 0.77 | .593 | 0.00 | 1.70 | .117 | 0.01 | 0.60 | .727 | 0.00 | 1.22 | .292 | 0.01 | 0.86 | .528 | 0.01 | 0.06 | .999 | 0.00 | 0.45 | 847 | 0.00 |
| Symptom Duration | 0.87 | .520 | 0.01 | 2.24 | .038 | 0.01 | 1.34 | .236 | 0.01 | 0.60 | .734 | 0.00 | 0.34 | .913 | 0.00 | 1.40 | .211 | 0.01 | 1.68 | .122 | 0.01 | 0.58 | .750 | 0.00 |
| Quality of life | 0.59 | .741 | 0.00 | 1.48 | .182 | 0.01 | 2.95 | .007 | 0.01 | 0.42 | .868 | 0.00 | 1.03 | .403 | 0.01 | 0.51 | .803 | 0.00 | 0.24 | .962 | .001 | 0.76 | .601 | .004 |
| Personal Control | 1.68 | .123 | 0.01 | 3.17 | .004 | 0.02 | 2.52 | .020 | .01 | 1.25 | .279 | 0.01 | 1.19 | .308 | 0.01 | 0.90 | .491 | 0.01 | 1.39 | .215 | 0.01 | 1.14 | .336 | 0.01 |
| Treatment Control | 1.90 | .077 | 0.01 | 2.08 | .053 | 0.01 | 1.61 | .140 | 0.01 | 1.10 | .362 | 0.01 | 1.53 | .166 | 0.01 | 0.58 | .747 | 0.00 | 1.61 | .140 | 0.01 | 1.01 | .417 | 0.01 |
| Illness Coherence | 6.72 | <.001 | 0.04 | 0.44 | .852 | 0.00 | 0.49 | .814 | 0.0 | 0.21 | .972 | 0.00 | 1.63 | .136 | 0.01 | 1.58 | .148 | 0.01 | 1.10 | .360 | 0.01 | 1.08 | .369 | 0.01 |

To assess the influence of gender, we ran a series 2 (illness description: Long COVID vs. ongoing COVID-19 recovery) X 2 (uncertainty: uncertainty emphasised vs. uncertainty not emphasised) X 2 (efficacy of support: enhanced support vs. basic support) X 2 (gender: woman vs. man) ANOVAs on symptom severity (consequences and emotional representation), symptom duration, quality of life, personal control, treatment control, and illness coherence. We only included women and men as there were not enough non-binary (*n*=6) for their own category. All results for these analyses are presented in Supplementary Table 4. We found a main effect of gender on emotional representation, symptom duration, personal control, and treatment control. Women reported higher emotional representation (p=.002, d=-0.19), longer symptom duration (p<.001, d=-0.20), but less personal control (p<.001, d=0.23) and less treatment control (p<.001, d=0.21) than men. There was also a significant interaction between illness uncertainty, efficacy of support, and gender on duration. First, in the uncertainty emphasised and enhanced support condition, women reported higher symptom duration than men (p=.002, d=-0.49). Second, men in the uncertainty emphasised and enhanced support condition reported less symptom duration than men (p<.001, d=-0.66) and women (p<.001, d=0.65) in the uncertainty not emphasises and enhances support condition, and men (<.001 d=-0.70) and women (p<.001, d=0.50) in the uncertainty not emphasised and basic support condition.

**Supplementary Table 4**

*The main effect and interacting effects of gender*

|  | Main Effect of Gender | | | Interaction Effect of Illness Description and Gender | | | Interaction Effect of and Illness Uncertainty and Gender | | | Interaction Effect of Efficacy of Support and Gender | | | Interaction Effect of Illness Description, Illness Uncertainty, and Gender | | | Interaction Effect of Illness Description, Efficacy of Support, and Gender | | | Interaction Effect of Illness Uncertainty, Efficacy of Support, and Gender | | | Interaction Effect of Illness Description,  Illness Uncertainty, Efficacy of Support, and Gender | | |
| --- | --- | --- | --- | --- | --- | --- | --- | --- | --- | --- | --- | --- | --- | --- | --- | --- | --- | --- | --- | --- | --- | --- | --- | --- |
|  | *F*(1, 692) | *p* | η_p_^2^ | *F*(1, 692) | *p* | η_p_^2^ | *F*(1, 692) | *p* | η_p_^2^ | *F*(1, 692) | *p* | η_p_^2^ | *F*(1, 692) | *p* | η_p_^2^ | *F*(1, 692) | *p* | η_p_^2^ | *F*(1, 692) | *p* | η_p_^2^ | *F*(1, 692) | *p* | η_p_^2^ |
| Symptom Severity: Consequences | 1.95 | .163 | 0.00 | 0.01 | .910 | 0.00 | 1.18 | .277 | 0.00 | 0.07 | .785 | 0.00 | 0.00 | .992 | 0.00 | 0.06 | .802 | 0.00 | 1.64 | .200 | 0.00 | 2.14 | .144 | 0.00 |
| Symptom Severity: Emotional Representation | 9.84 | .002 | 0.01 | 0.14 | .713 | 0.00 | 0.79 | .373 | 0.00 | 0.12 | .729 | 0.00 | 0.01 | .912 | 0.00 | 0.23 | .631 | 0.00 | 0.49 | .485 | 0.00 | 1.53 | .216 | 0.00 |
| Symptom Duration | 11.45 | <.001 | 0.01 | 0.38 | .540 | 0.00 | 2.66 | .103 | 0.00 | 0.57 | .449 | 0.00 | 0.22 | .642 | 0.00 | 0.09 | .764 | 0.00 | 5.33 | .021 | 0.01 | 0.75 | .385 | 0.00 |
| Quality of life | 2.55 | .111 | 0.00 | 0.17 | .677 | 0.00 | 0.47 | .494 | 0.00 | 0.15 | .702 | 0.00 | 0.52 | .470 | 0.00 | 0.00 | .953 | 0.00 | 1.24 | .266 | 0.00 | 1.20 | .274 | 0.00 |
| Personal Control | 14.74 | <.001 | 0.01 | 0.80 | .373 | 0.00 | 1.85 | .175 | 0.00 | 0.02 | .882 | 0.00 | 0.37 | .545 | 0.00 | 1.43 | .231 | 0.00 | 0.18 | .672 | 0.00 | 2.37 | .124 | 0.00 |
| Treatment Control | 12.28 | <.001 | 0.01 | 1.54 | .215 | 0.00 | 0.64 | .422 | 0.00 | 0.18 | .673 | 0.00 | 1.30 | .254 | 0.00 | 3.55 | .060 | 0.00 | 0.19 | .666 | 0.00 | 3.51 | .061 | 0.00 |
| Illness Coherence | 1.19 | .276 | 0.00 | 0.58 | .447 | 0.00 | 2.07 | .150 | 0.00 | 0.72 | .395 | 0.00 | 0.04 | .848 | 0.00 | 2.39 | .122 | 0.00 | 0.13 | .716 | 0.00 | 0.63 | .427 | 0.00 |
